# Safety and Efficacy of Immune Checkpoint Inhibitors in Patients with Cancer and Viral Hepatitis: The MD Anderson Cancer Center Experience

**DOI:** 10.1093/oncolo/oyad039

**Published:** 2023-03-23

**Authors:** Mirella Nardo, Bulent Yilmaz, Blessie Elizabeth Nelson, Harrys A Torres, Lan Sun Wang, Bruno Palma Granwehr, Juhee Song, Hanna R F Dalla Pria, Van A Trinh, Isabella C Glitza Oliva, Sapna P Patel, Nizar M Tannir, Ahmed Omar Kaseb, Mehmet Altan, Sunyoung S Lee, Ethan Miller, Hao Zhang, Bettzy A Stephen, Aung Naing

**Affiliations:** Department of Investigational Cancer Therapeutics, The University of Texas MD Anderson Cancer Center, Houston, TX, USA; Department of Investigational Cancer Therapeutics, The University of Texas MD Anderson Cancer Center, Houston, TX, USA; Department of Investigational Cancer Therapeutics, The University of Texas MD Anderson Cancer Center, Houston, TX, USA; Department of Infectious Diseases Infection Control and Employee Health, The University of Texas MD Anderson Cancer Center, Houston, TX, USA; Department of Genitourinary Medicine, The University of Texas MD Anderson Cancer Center, Houston, TX, USA; Department of Infectious Diseases Infection Control and Employee Health, The University of Texas MD Anderson Cancer Center, Houston, TX, USA; Department of Biostatistics, The University of Texas MD Anderson Cancer Center, Houston, TX, USA; Department of Radiology, The University of Texas MD Anderson Cancer Center, Houston, TX, USA; Department of Melanoma Medicine, The University of Texas MD Anderson Cancer Center, Houston, TX, USA; Department of Melanoma Medicine, The University of Texas MD Anderson Cancer Center, Houston, TX, USA; Department of Melanoma Medicine, The University of Texas MD Anderson Cancer Center, Houston, TX, USA; Department of Genitourinary Medicine, The University of Texas MD Anderson Cancer Center, Houston, TX, USA; Department of Genitourinary Medicine, The University of Texas MD Anderson Cancer Center, Houston, TX, USA; Department of Genitourinary Medicine, The University of Texas MD Anderson Cancer Center, Houston, TX, USA; Department of Thoracic/Head and Neck Medical Oncology, The University of Texas MD Anderson Cancer Center, Houston TX, USA; Department of Gastrointestinal Medicine, The University of Texas MD Anderson Cancer Center, Houston, TX, USA; Department of Gastrointestinal Medicine, The University of Texas MD Anderson Cancer Center, Houston, TX, USA; Department of Gastrointestinal Medicine, The University of Texas MD Anderson Cancer Center, Houston, TX, USA; Department of Investigational Cancer Therapeutics, The University of Texas MD Anderson Cancer Center, Houston, TX, USA; Department of Investigational Cancer Therapeutics, The University of Texas MD Anderson Cancer Center, Houston, TX, USA

**Keywords:** immune checkpoint inhibitors, advanced cancer, viral hepatitis, advanced liver disease, safety

## Abstract

**Background:**

Despite the clinical benefit of immune checkpoint inhibitors (ICIs), patients with a viral hepatitis have been excluded from clinical trials because of safety concerns. The purpose of this study was to determine the incidence rate of adverse events (AEs) in patients with viral hepatitis who received ICIs for cancer treatment.

**Materials and Methods:**

We conducted a retrospective study in patients with cancer and concurrent hepatitis B or C, who had undergone treatment with ICI at MD Anderson Cancer Center from January 1, 2010 to December 31, 2019.

**Results:**

Of the 1076 patients screened, we identified 33 with concurrent hepatitis. All 10 patients with HBV underwent concomitant antiviral therapy during ICI treatment. Sixteen of the 23 patients with HCV received it before the initiation of ICI. The median follow-up time was 33 months (95% CI, 23-45) and the median duration of ICI therapy was 3 months (IQR, 1.9-6.6). Of the 33 patients, 12 (39%) experienced irAEs (immune-related adverse events) of any grade, with 2 (6%) having grade 3 or higher. None of the patients developed hepatitis toxicities.

**Conclusion:**

ICIs may be a therapeutic option with an acceptable safety profile in patients with cancer and advanced liver disease.

## Teaser

Despite the clinical benefit with immune checkpoint inhibitors (ICIs), patients with a viral hepatitis have been excluded from clinical trials because of safety concerns. This article reports on incidence rate of adverse events in patients with viral hepatitis who received ICIs for cancer treatment.

**Implications for Practice**

This article reports the institutional experience with the treatment of patients with viral hepatitis and advanced hepatic disease treated with immune checkpoint inhibitors outside a clinical trial setting at MD Anderson Cancer Center. There is no robust evidence for treating this population in regular clinical settings due to a lack of information on the safety and efficacy of immunotherapeutic agents in these patients as they are often excluded from clinical trials. As immunotherapies’ indications broaden, we sense an urgency to disseminate this information to the healthcare professionals in the community.

## Introduction

Treatment strategies with immune checkpoint inhibitors (ICIs) have shown promising survival benefits^1–3^ and have been rapidly approved to treat different tumor types in recent years. Despite these encouraging results, patients with a concurrent diagnosis of viral hepatitis have been excluded from the majority of ICI-based clinical trials because of safety concerns regarding possible hepatic toxicity and viral reactivation.^4,5^ Only a few studies of ICIs have included patients with viral hepatitis and cancer, and those studies used restrictive patient selection criteria; therefore, the published data on the safety and efficacy of ICIs in this patient population is limited to those with a low viral load, preserved hepatic function, and an ideal performance status.

In 2013, a prospective study evaluated the use of tremelimumab, a cytotoxic T-lymphocyte-associated antigen 4 inhibitor, in 22 patients with chronic hepatitis C virus (HCV) infection and hepatocellular carcinoma (HCC).^6^ The results showed some transient elevation of transaminases but no HCV infection exacerbation or hepatitis flare.

In another retrospective study of 40 patients with active or resolved HCV, who received ICI for cancer treatment, only 2 had ≥ grade 3 immune-related adverse events (irAEs) (colitis and pneumonitis). Two patients experienced hepatotoxicity, one had grade 1 and the other grade 2. The patient with grade 2 hepatotoxicity was able to resume ICI after a short course of steroids. In general, the AE profile was comparable to the published data in non-HCV patients. There were no deaths as a complication of ICIs.^7^

In the CheckMate-040 trial,^8^ among all of the patients with advanced HCC who were treated with nivolumab, a programmed cell death protein-1 inhibitor, 51 had HBV and 50 had HCV; no patients were found to have signs of hepatitis reactivation. Patients were required to have a viral load less than 100 IU/mL for HBV and they were eligible only if they had a Child-Pugh score of 6 or lower (Child-Pugh A) and an Eastern Cooperative Oncology Group performance status of 1 or lower, for the dose-expansion cohort.

The KEYNOTE-224 study^9^ evaluated 22 patients with advanced HCC and HBV and 26 with HCV who had been treated with pembrolizumab, a programmed cell death ­protein-1 inhibitor. No HBV or HCV flares occurred. All patients were Child-Pugh class A and had an Eastern Cooperative Oncology Group performance status of 0-1.

In the CheckMate 459 study,^10^ nivolumab was compared to sorafenib in the first-line setting for HCC. Patients with viral hepatitis were included; however, patients with chronic HBV infection were required to receive antiviral therapy and have a viral load of <100 IU/mL. Patients with HCV were excluded if they met the criteria to receive antiviral treatment. Among this selected population, there was no virus reactivation reported, but 3 patients in the nivolumab group and 1 patient in the sorafenib group died due to hepatotoxicity, considered related to the drugs. There was no available information regarding their viral hepatitis diagnosis and status.

In this study, we determined the incidence rate of hepatitis reactivation, hepatic toxicity, and liver failure in patients with concurrent viral hepatitis and advanced liver disease who received ICIs for cancer treatment in a non-clinical trial setting. We also described the efficacy assessment regarding treatment outcomes in this population (overall response rate [ORR] and progression-free survival [PFS] and overall survival [OS] durations).

## Methods

This retrospective study was approved by the Institutional Review Board (IRB) at The University of Texas MD Anderson Cancer Center, Houston, TX, USA (Protocol PA15-0798). Study subjects were identified by the institution’s medical record number and their privacy was protected according to institutional and HIPAA guidelines.

The requirement to obtain informed consent was waived due to the retrospective nature of the study. This retrospective study was conducted in accordance with the Declaration of Helsinki and the International Conference on Harmonization Good Clinical Practice guidelines.

### Patients

We used the pharmacy list of all patients with cancer who had undergone treatment with a commercial supply of any ICI at MD Anderson from January 1, 2010 to December 31, 2019, and who had been screened for viral hepatitis, which was defined as having a positive HBsAg (in the presence of positive hepatitis B core antibody) or viral load for hepatitis B, and a positive anti-HCV confirmed by the current positive viral load for hepatitis C. We reviewed the institutional electronic medical records and collected the demographic and clinical data of the patients with a confirmed viral hepatitis diagnosis.

### Procedures

Adverse events (AEs) that were definitively, probably, or possibly related to ICIs were graded according to the National Cancer Institute Common Terminology Criteria for Adverse Events version v5.0 (CTCAE v5.0, U.S. Department of Health and Human Services).^11^

To investigate complications related to the ICI treatment concurrent to the viral hepatitis diagnosis, we assessed ALT (alanine transaminase), AST (aspartate aminotransferase), INR (international normalized ratio based on prothrombin time), and total bilirubin levels, and cirrhosis evaluation scores (APRI, FIB-4, Child, and MELD). All these information were collected before, during (each oncology visit), and after ICI treatment, when these variables were available.

We applied the American Association for the Study of Liver Diseases Guidance^12,13^ to determine the occurrence of hepatitis flare or hepatic failure. In addition, we collected data on the viral load (RNA or DNA), antigens (HBsAg and HBeAg), and antibodies (anti-HCV, anti-HBsAg, anti-HbeAg, and anti-HBcAg) that were associated with hepatitis diagnosis before, during, and after ICI treatment, when available in the records. We also investigated any evidence of viral hepatitis reactivation.^12,14^

We extracted data on confounding factors such as comorbidities, presence of neoplastic liver lesions, signs of biliary obstruction, administration of chemotherapeutic agents 6 months prior to ICI therapy, previous treatment for hepatitis with antivirals, use of steroids before, during, and after ICI therapy, acute infections or other complications not related to ICI therapy, and, concomitant use of hepatotoxic drugs.^15^

Investigators in the Department of Radiology at MD Anderson performed a retrospective evaluation of the available CT images that were obtained at baseline and during ICI therapy. Response to ICI treatment was measured according to the Response Evaluation Criteria in Solid Tumors (RECIST) 1.1^16^ and irRECIST.^17^

### Objectives and Statistical Analysis

The primary objective of our study was to evaluate the safety and tolerability of ICI treatment in cancer patients with chronic viral hepatitis, as determined by the incidence rates of hepatitis reactivation, hepatic toxicity, and liver failure.

The secondary objectives were to evaluate ORR,^14^ disease control rate (DCR), PFS, and OS. The best overall response was defined as the best response observed from the start of the treatment until disease progression or discontinuation of treatment for any reason. ORR was defined as the percentage of patients whose best overall response was a confirmed complete response or partial response. DCR was defined as percentage of patients whose best overall response was either objective response or stable disease. PFS was defined as the time interval from the initiation of ICI therapy to the first documented tumor progression or death from any cause. Patients who were alive and had no disease progression or who were lost to follow-up were censored at the last imaging assessment. OS was defined as the time interval from the initiation of ICI therapy to death from any cause. Patients who were alive or lost to follow-up were censored at the time of the last known follow-up.

Patient characteristics were summarized for all patients using descriptive statistics, mean (SD) or median (IQR) for continuous variables, and frequency (%) for categorical variables. The incidence rate of hepatitis reactivation, hepatic toxicity, and liver failure was estimated along with their 95% CIs. The incidence rate of the AEs and 95% CI were estimated. The ORR, CBR, and associated 2-sided 95% CI were estimated. A waterfall plot was used to illustrate the maximum percentage of change in tumor measurements from baseline per irRECIST. The median PFS and OS were summarized by the Kaplan-Meier method. SAS 9.4 (SAS Institute, Inc., Cary, NC, USA) was used for data analysis.

## Results

### Patients

**Figure 1.**
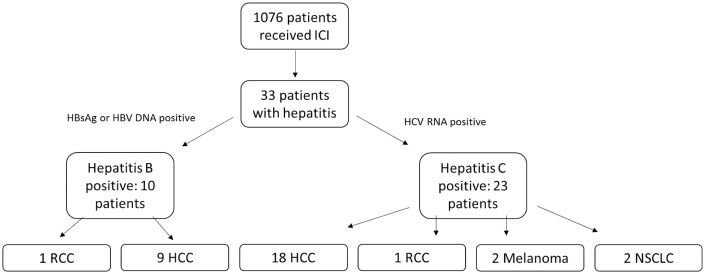
CONSORT diagram. *Abbreviations: HBsAg, hepatitis B surface antigen; HCC, hepatocellular carcinoma; ICI, immune checkpoint inhibitor; NSCLC, non-small cell lung cancer; RCC, renal cell carcinoma.

We identified 1076 patients who had undergone ICI treatment; 33 had a viral hepatitis diagnosis prior to treatment and were included in this study (Fig. 1). Among the 33 patients, 10 had HBV and 23 had HCV at the date of ICI initiation. The median age at ICI initiation was 61 years, and 27 patients were male. All of the 33 patients received anti-PD1/anti-PD-L1, either alone (27), or combined with TACE (transarterial chemoembolization = 2), bevacizumab (1), chemotherapy (carboplatin plus pemetrexed = 1), or anti-CTLA4 (2). Patients baseline characteristics are summarized in Table 1.

**Table 1.** Baseline characteristics of patients with solid tumors and chronic viral hepatitis receiving ICIs (*n* = 33).

Characteristic	Count (%)
Median age ± SD, years	61.0 ± 8.7
Sex	
Male	27 (82%)
Female	6 (18%)
Eastern Cooperative Oncology Group	
0	8 (24%)
1	19 (58%)
2	3 (9%)
NA	3 (9%)
Solid tumor type	
Hepatocellular carcinoma	27 (82%)
Lung adenocarcinoma	2 (6%)
Melanoma	2 (6%)
Renal cell carcinoma	2 (6%)
Cancer stage	
III	5 (15%)
IV	28 (85%)
Hepatitis diagnosis	
B	10 (30%)
C	23 (70%)
History of alcohol intake	
Positive	11 (33%)
Negative	21 (64%)
NA	1 (3%)
Cirrhosis	
Yes	28 (85%)
No	5 (15%)
Child-Pugh score	
5	18 (55%)
6	5 (15%)
7	3 (9%)
8	2 (6%)
NA	5 (15%)
Liver primary lesion/metastasis	
Yes	28 (85%)
No	5 (15%)
Number of prior lines	
0	4 (12%)
1	24 (73%)
2	2 (6%)
≥3	3 (9%)
Median number of comorbidities (range)	2 (range 0-7)
ICI therapy type	
Anti-PD-1 monotherapy	31 (94%)
Anti-PD-1 with anti-CTLA-4	2 (6%)
Anti-viral treatment	
Yes	26 (79%)
No	7 (21%)
HBV treatment concomitant to ICIs	10 (100%)
HCV treatment^*^	16 (70%)
Interferon based^*^	6 (26%)
DAA based^*^	10 (43%)

Abbreviations: CTLA-4, cytotoxic T-lymphocyte-associated antigen 4; DAA, direct-acting antiviral; NA, not available; PD-1, programmed cell death protein-1.

^*^Among patients with HCV.

All of the patients were classified as having advanced disease, 5 with stage III and 28 with stage IV. The most common diagnosis was HCC (*n* = 27), followed by non-small cell lung cancer, melanoma, and renal cell carcinoma (*n* = 2 each).

Among the 33 patients, 28 (85%) had been diagnosed with cirrhosis before starting ICIs. Five had a Child-Pugh score of 7 or higher, and 1 had a history of hepatitis flare, which was controlled before starting ICIs. In the 6 months prior to ICI treatment, 31 patients received drugs that were associated with a medium risk of viral hepatitis reactivation,^14^ but no patients received medication that was associated with a high risk of viral hepatitis reactivation.

Among patients with hepatitis B and C, the complete data on viral load was not available for all patients. The baseline serologies of patients with hepatitis B and C are described in Tables 2 and 3, respectively. The upper limit of normal for ALT and AST in our laboratory is 41 and 40 U/L, respectively. The average ALT and AST among the patients with hepatitis B was 94.9 U/L (range: 15-203) and 63.7 U/L (range: 24-163), respectively. The average among patients with hepatitis C was ALT 53.2 U/L (range: 20-103) and AST 83.8 U/L (range: 15-232).

**Table 2.** Viral hepatitis status and anti-viral treatment among patients with hepatitis B (*n* = 10).

HBsAg/HBeAg/HBV DNA (IU/mL)	Known cirrhosis	ALT (U/L)	AST (U/L)	Anti-viral treatment	Time between starting antiviral and ICI (months)
HBsAg+/HBeAg uk/HBV DNA undetectable	Y	15	29	tenofovir	79
HBsAg+/HBeAg neg/HBV DNA 58	N	29	30	entecavir	NA
HBsAg uk/HBeAg uk/HBV DNA detected	Y	36	24	entecavir	28
HBsAg+/HBeAg neg/HBV DNA detected	Y	82	80	tenofovir	NA
HBsAg+/HBeAg neg/HBV DNA 1580 000	Y	55	42	entecavir	12
HBsAg+/HBeAg neg/HBV DNA detected	Y	70	57	entecavir	NA
HBsAg+/HBeAg+/HBV DNA <10	Y	217	163	tenofovir	345
HBsAg+/HBeAg neg/HBV DNA 24	Y	106	116	tenofovir	5
HBsAg+/HBeAg neg/HBV DNA undetectable	Y	136	56	entecavir	NA
HBsAg+/HBeAg neg/HBV DNA undetectable	Y	203	40	entecavir	89

Abbreviations: NA, non-available; uk, unknown.

**Table 3.** Viral hepatitis status and anti-viral treatment among patients with hepatitis C (*n* = 23).

Anti-HCV and HCV RNA (IU/ml))	Known cirrhosis	ALT (U/L)	AST (U/L)	Anti-viral treatment	Time between starting antiviral and ICI (months)
Anti-HCV+/RNA undetectable	Y	64	87	IFN + Ribavirin	234
Anti-HCV+/no RNA not available	Y	42	70	N	NA
Anti-HCV+/no RNA not available	Y	30	56	Ledipasvir-Sofosbuvir	NA
Anti-HCV+/RNA undetectable	Y	32	16	Ledipasvir-Sofosbuvir	18
Anti-HCV/RNA: 1800 000	Y	30	84	N	
Anti-HCV+/no RNA not available	Y	28	63	Ledipasvir-Sofosbuvir	28
Anti-HCV+/RNA: 812 000	Y	51	119	Ledipasvir-Sofosbuvir	18
Anti-HCV+/RNA undetectable	Y	50	61	Ledipasvir-Sofosbuvir	37
Anti-HCV+/RNA: 149 000	N	103	97	N	NA
Anti-HCV+/RNA 954 000	Y	139	250	IFN + Ledipasvir-Sofosbuvir	44
Anti-HCV+/RNA undetectable	Y	62	80	Ledipasvir-Sofosbuvir	13
Anti-HCV+/RNA 25 700	Y	69	101	Ledipasvir-Sofosbuvir	9
Anti-HCV+/RNA undetectable	Y	72	232	IFN + Ribavirin + Boceprevir	NA
Anti-HCV+/RNA undetectable	Y	41	39	Elbasvir-Grazoprevir	NA
	Y	48	46	N	NA
Anti-HCV+/RNA not available	N	30	33	IFN	NA
Anti-HCV+/RNA undetectable	Y	44	70	Sofosbuvir-Velpatasvir	24
Anti-HCV+/RNA undetectable	Y	38	100	Ledipasvir-Sofosbuvir	55
Anti-HCV+/RNA undetectable	N	20	15	N	NA
Anti-HCV+/RNA: 190 000	Y	27	63	N	NA
Anti-HCV+/RNA undetectable	Y	37	42	IFN + Ledipasvir-Sofosbuvir	NA
Anti-HCV+/no RNA not available	Y	27	97	NA	7
Anti-HCV+/RNA undetectable	N	140	107	IFN	NA

Abbreviation: NA, non-available.

All 10 patients with HBV underwent concomitant antiviral treatment. Sixteen of the 23 patients with HCV received anti-viral treatment before the initiation of ICI. Six of these 16 patients had previously received interferon, corresponding to 26% of the patients with hepatitis C. The average period between the initiation of anti-viral treatment and ICI among the patient with hepatitis B was 93 months (range: 5-345). The average between the anti-viral treatment and the initiation of ICI among patients with hepatitis C was 44.2 months (range: 7-234).

### Safety

The median follow-up time was 33 months (95% CI, 23-45). The median duration of ICI therapy was 3 months (IQR, 1.9-6.6). Of the 33 patients, 12 (39%) experienced irAEs of any grade (Table 4). Two (6%) patients had grade 3 or higher irAEs. One had a grade 3 lichenoid eruption, and another had a grade 3 pneumonitis, leading to ICI discontinuation. Treatment was discontinued in 2 other patients due to grade 2 diarrhea and grade 2 osteoarthritis, resulting in the termination of ICI treatment due to irAEs in 12% of patients.

**Table 4.** irAEs according to common terminology criteria for adverse events v5.0.

irAE	Any grade	Grade ≥3
Any	12 (39%)	2 (6%)
Rash	6 (18%)	0 (0%)
Diarrhea	2 (6%)	0 (0%)
Hypothyroidism	1 (3%)	0 (0%)
Arthritis	1 (3%)	0 (0%)
Lichenoid eruptions	1 (3%)	1 (3%)
Pneumonitis	1 (3%)	1 (3%)

During ICI treatment, 4 patients (12%) required steroids, but only 3 of them received it due to irAE. One patient who had RSV bronchiolitis (consequently, considered not related to the ICI), received 3 weeks of steroids and resumed the ICI without any new adverse events or viral hepatitis reactivation. Of the 3 patients requiring systemic steroids for the management of irAEs, the patient with pneumonitis had disease progression and sepsis, resulting in death in the next few weeks, not considered as a consequence of irAE. The 2nd patient had a grade 2 osteoarthritis exacerbation in knees and hips, which resolved after systemic and intra-articular steroids, besides the discontinuation of the ICI treatment. The 3rd patient had a grade 2 colitis, which was partially controlled with steroids but was lost to follow up in the subsequent weeks. The 2nd and 3rd patient had a stable disease and partial response as their best response, respectively.

The patient with grade 2 osteoarthritis exacerbation, who had a documentation on viral load before, at the end of tapering of steroids, and 1 year after the steroid treatment, showed undetectable HCV RNA consistently. This patient had completed his treatment with a direct-acting antiviral combination 17 months prior to receiving systemic steroids. Stable disease was documented for 2 years after ICI discontinuation despite being without any treatment. Currently, the patient is alive after almost 4 years since ICI discontinuation.

None of the 33 patients developed hepatitis reactivation, hepatitis flare, hepatic failure, or any sign of immune-­mediated hepatotoxicity during or up to 6 months after ICIs. There was also no documentation of viral hepatitis reactivation among those who received steroids.

There were 8 AEs that were ICI-related, but not immune-mediated. They were fatigue (*n* = 4), anorexia (*N* = 3), and nausea (*N* = 1). None of these AEs were ≥grade 3.

### Efficacy

**Figure 2.**
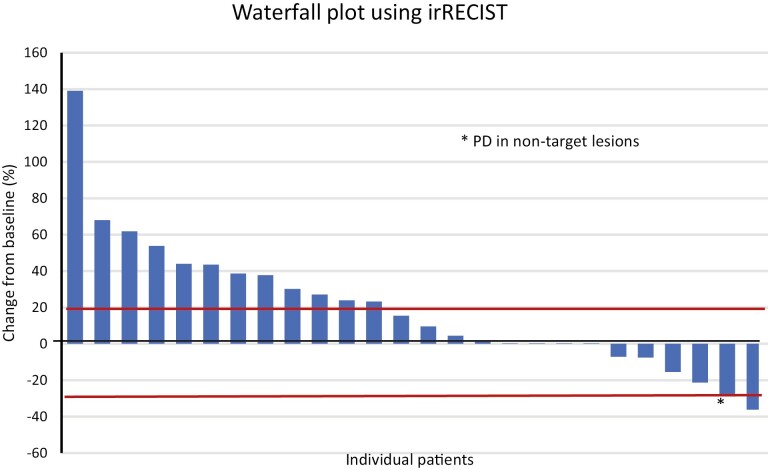
Waterfall plot using irRECIST in patients with hepatocellular cancer with chronic viral hepatitis (*n* = 27).

For the efficacy evaluation, we included only patients with a diagnosis of HCC who had received at least 1 cycle of ICI. Among the 27 patients with HCC, 1 patient had a partial response for an ORR of 3.7% and 12 additional patients had stable disease for a DCR of 51.8% as described in Table 5. The best overall responses seen in these 27 patients are illustrated in Fig. 2.

**Table 5.** Best response by RECIST 1.1 and irRECIST in patients with hepatocellular cancer with chronic viral hepatitis (*n* = 27).

Best overall response	*N* (%)
Complete response	0
Partial response	1 (3.7)
Stable disease	12 (44.4)
Progression of disease	13 (48.1)
Not evaluable	1 (3.7)

**Figure 3.**
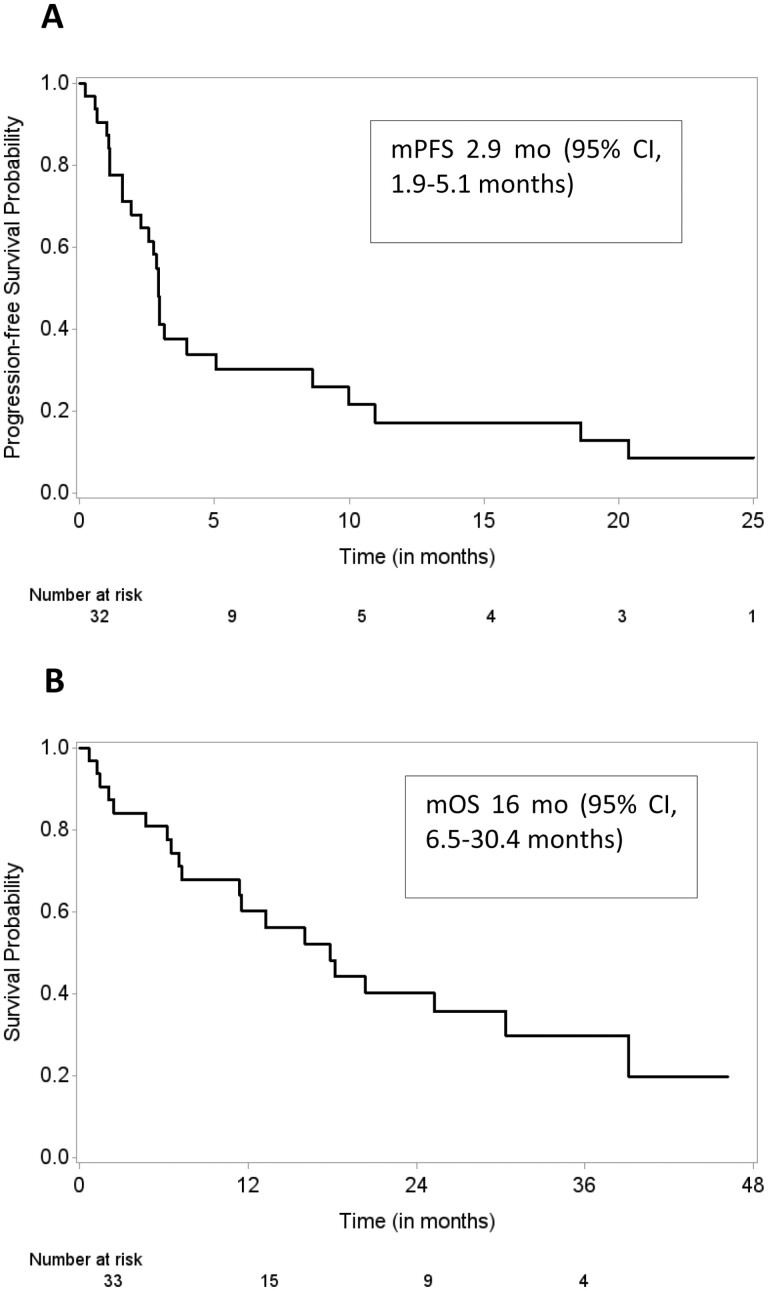
Survival outcomes in 27 patients with hepatocellular carcinoma with chronic viral hepatitis on treatment with immune checkpoint inhibitor. (**A**) Progression-free survival and (**B**) overall survival.

The median PFS was 2.9 months (95% CI, 1.9-4.0 months), and the median OS was 16 months (95% CI, 6.5-30.4 months), as illustrated in Fig. 3A, 3B, respectively.

## Discussion

While ICIs have revolutionized the treatment landscape of several cancers, patients with chronic hepatitis are often excluded from receiving immunotherapy due to potential risk for viral reactivation.^5,18–20^ In addition, conflicting data on efficacy of ICIs to reinvigorate the T cells that are in a state of exhaustion due to persistent viral stimulation has dampened research in this area.^21^ As the available evidence on the safety and efficacy of ICIs in this high-risk population is limited, the key question, “Will this therapy lead to further immune dysregulation in those patients who are already at higher risk for autoimmunity?” still remains.^4^

To address this question in patients with advanced cancer and chronic hepatitis, we screened 1076 consecutive patients who received commercial supply of ICI at MD Anderson for treatment of cancer. We noted that only 3% of the patients that received ICI in non-clinical trial setting had concurrent chronic hepatitis. This may be an under representation due to the limited use of ICIs given the lack of evidence on the safety and efficacy of immunotherapeutic agents in this high-risk population.

The rate of grade 3 or more treatment-related AEs in our study was 6%. The low incidence rate of treatment-related AEs in our study is similar to that found in a meta-­analyses of 22 ICI-based clinical trials^22^ reporting patients with advanced or metastatic solid-organ malignancies, where patients receiving ICI-based therapy had lower AE rates than those receiving chemotherapy. Furthermore, none of the patients had hepatitis reactivation, hepatitis flare, or hepatic failure during ICI therapy. This suggests that ICIs may be a safe therapeutic option in patients with chronic hepatitis. However, caution must be exercised as irAEs associated with ICIs can be potentially fatal if not treated promptly. In our study, adverse events led to the discontinuation of ICIs in 4 patients.

Nevertheless, irAEs could be associated with response to treatment with ICIs^23^ and patients are known to derive benefit from ICIs even after discontinuation of ICI due to irAEs.^24^ Consistent with published data that disease control or survival benefits are not affected by the occurrence of irAEs or the need for systemic corticosteroids for management of irAEs,^25,26^ in our study we observed that patients with grade 2 osteoarthritis and colitis had disease control even after systemic use of steroids for management of irAEs. The patient with grade 2 osteoarthritis continued to have survival benefits 4 years after the treatment with ICI was stopped.

In our study, 3.7% of patients had an objective responses and 48.1% had disease control. Few clinical trials have reported preliminary evidence of antitumor activity of ICIs in cancer patients with chronic viral hepatitis, and they commonly excluded patients with documented cirrhosis and advanced liver disease. For example, in CheckMate 459,^10^ a phase 3 trial of nivolumab as first-line therapy for advanced hepatocellular carcinoma, 19% of HBV infected and 17% of HCV-infected patients had objective response compared to 12% in uninfected patients. Similar results were also reported in CheckMate 040^8^ study of nivolumab in patients with advanced HCC. Objective response was reported in 14% of HBV infected and 20% of HCV infected patients and clinical benefit in 55% and 66% of patients respectively. No patients had viral reactivation and adverse events were similar in patients with or without hepatitis.

The strength of our study is that we aimed to evaluate patients in a non-clinical trial setting, not only with viral hepatitis diagnosis but also with an advanced liver disease/cirrhosis (almost 50% had a Child-Pugh score of 6 or more), multiple comorbidities, and prior lines of treatment (only 12% were treatment naive). We found that ICIs in patients with chronic viral hepatitis (28 with advanced cirrhosis) and several comorbidities (median of 2) had an acceptable safety profile and none of the 33 patients developed hepatitis reactivation, hepatitis flare, or hepatic failure due to the treatment. Although this could be attributed in part to the anti-viral treatment they received for hepatitis based on evaluation by a hepatologist before starting the ICI treatment, our findings lend support to the hypothesis from earlier studies that ICIs could attenuate hepatitis B infection by activation of T-cell response, further implying that we may not expect viral hepatitis B flares.^27^ In this context, the need for systemic steroids and immunosuppressors for the treatment of irAEs may be the biggest concern for the risk of viral reactivation. However, in our study, none of the 4 patients who received corticosteroids had a viral hepatitis reactivation or hepatitis flare. Further, one patient had a documented undetectable viral load a year after prolonged steroid use, suggesting that a history of chronic hepatitis and systemic use of steroids for the management of irAEs may not preclude this patient population from receiving ICI treatment. As it is unknown if a similar response and toxicity profile can be seen in patients with hepatitis B who have not received concurrent antivirals, it may be worth investigating if the practice of treating cancer patients with chronic viral hepatitis with antivirals prior to treatment with ICIs should become a standard of care, aligned to the current ASCO recommendation for hepatitis B virus screening and treatment before cancer treatment.^20^

There is no robust prospective data on ICI use in patients with advanced liver disease because the majority of previous studies^6,8–10^ required a Child-Pug class A, Eastern Cooperative Oncology Group performance status score of 0-1, adequate organ function, and stable hepatic disease status as evidenced by no history of hepatic encephalopathy, clinically significant ascites, and portal hypertension. Considering the possible benefit, we believe that these high-risk patients could be more widely included in future clinical trials.^28^

There are several limitations to our study. Considering the small sample and the retrospective nature, we acknowledge the possible biases, specifically regarding the documentation of the adverse events and availability of laboratory measures. Nevertheless, our study demonstrates that ICIs may be a therapeutic option with an acceptable safety profile and antitumor activity that could be administered with close monitoring in patients with cancer and advanced liver disease due to chronic hepatitis, which warrants further investigation.

## Data Availability

The datasets generated during and/or analyzed during the current study are available from the corresponding author on reasonable request.
