# Correction to: Safety and Efficacy of Immune Checkpoint Inhibitors in Patients with Cancer and Viral Hepatitis: The MD Anderson Cancer Center Experience

**DOI:** 10.1093/oncolo/oyad142

**Published:** 2023-05-15

This is a correction to: Mirella Nardo, Bulent Yilmaz, Blessie Elizabeth Nelson, Harrys A Torres, Lan Sun Wang, Bruno Palma Granwehr, Juhee Song, Hanna R F Dalla Pria, Van A Trinh, Isabella C Glitza Oliva, Sapna P Patel, Nizar M Tannir, Ahmed Omar Kaseb, Mehmet Altan, Sunyoung S Lee, Ethan Miller, Hao Zhang, Bettzy A Stephen, Aung Naing, Safety and Efficacy of Immune Checkpoint Inhibitors in Patients with Cancer and Viral Hepatitis: The MD Anderson Cancer Center Experience, *The Oncologist*, 2023; oyad039, https://doi.org/10.1093/oncolo/oyad039

In the originally published version of this manuscript, Mehmet Altan’s affiliation was presented incorrectly. The correct affiliation for this author is: Department of Thoracic/Head and Neck Medical Oncology, The University of Texas MD Anderson Cancer Center, Houston TX, USA.

This error has been corrected online.

